# (Nitrato-κ^2^ *O*,*O*′)bis­[(*E*)-*N*-(pyridin-4-yl­methyl­idene-κ*N*)hy­droxy­amine]­silver(I)

**DOI:** 10.1107/S1600536812046107

**Published:** 2012-11-28

**Authors:** Shan Gao, Seik Weng Ng

**Affiliations:** a Key Laboratory of Functional Inorganic Material Chemistry, Ministry of Education, Heilongjiang University, Harbin 150080, People’s Republic of China; b Department of Chemistry, University of Malaya, 50603 Kuala Lumpur, Malaysia; c Chemistry Department, Faculty of Science, King Abdulaziz University, PO Box 80203 Jeddah, Saudi Arabia

## Abstract

In the mononuclear title compound, [Ag(NO_3_)(C_6_H_6_N_2_O)_2_], the Ag^I^ atom is located on a twofold rotation axis and the nitrate-chelated Ag^I^ atom is further coordinated by two aromatic N atoms of hydroxyl­amine ligands in a distorted tetra­hedral geometry. In the crystal, the nitrate ion has 2 symmetry with the N atom and one O atom located on the twofold rotation axis, and is linked to hy­droxy groups of the hydroxyl­amine ligands by O—H⋯O hydrogen bonds, generating a chain running along the *b* axis.

## Related literature

For (nitrato)(picolinaldehyde oxime)silver(I), see: Abu-Youssef *et al.* (2010 ▶).
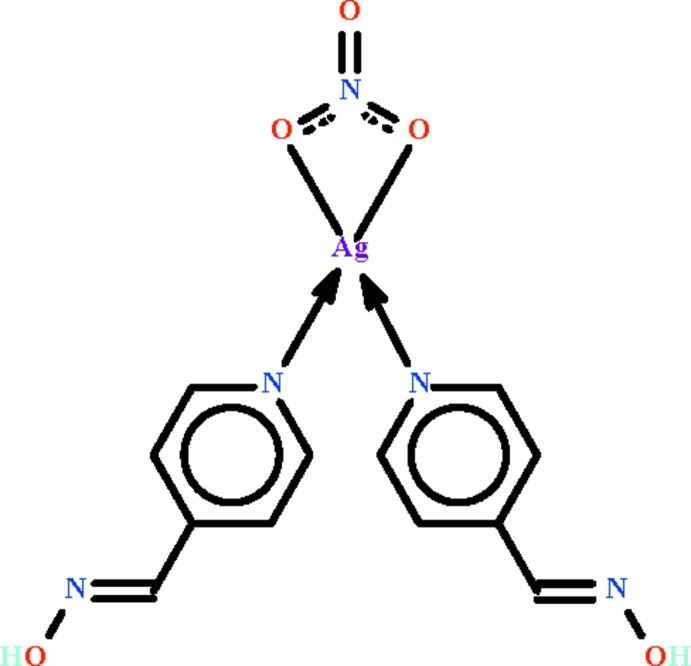


## Experimental

### 

#### Crystal data

[Ag(NO_3_)(C_6_H_6_N_2_O)_2_]
*M* *_r_* = 414.14
Orthorhombic, 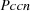

*a* = 18.027 (3) Å
*b* = 4.6907 (6) Å
*c* = 17.7020 (19) Å
*V* = 1496.9 (3) Å^3^
*Z* = 4
Mo *K*α radiation
μ = 1.38 mm^−1^
*T* = 293 K
0.20 × 0.12 × 0.12 mm

#### Data collection

Rigaku R-AXIS RAPID IP diffractometer
Absorption correction: multi-scan (*ABSCOR*; Higashi, 1995 ▶) *T*_min_ = 0.770, *T*_max_ = 0.852
13223 measured reflections
1705 independent reflections
1038 reflections with *I* > 2σ(*I*)
*R*_int_ = 0.082

#### Refinement

*R*[*F* ^2^ > 2σ(*F* ^2^)] = 0.050
*wR*(*F* ^2^) = 0.162
*S* = 1.13
1705 reflections
108 parameters
H-atom parameters constrained
Δρ_max_ = 0.92 e Å^−3^
Δρ_min_ = −0.67 e Å^−3^

### 

Data collection: *RAPID-AUTO* (Rigaku, 1998 ▶); cell refinement: *RAPID-AUTO*; data reduction: *CrystalClear* (Rigaku/MSC, 2002 ▶); program(s) used to solve structure: *SHELXS97* (Sheldrick, 2008 ▶); program(s) used to refine structure: *SHELXL97* (Sheldrick, 2008 ▶); molecular graphics: *X-SEED* (Barbour, 2001 ▶); software used to prepare material for publication: *publCIF* (Westrip, 2010 ▶).

## Supplementary Material

Crystal structure: contains datablock(s) global, I. DOI: 10.1107/S1600536812046107/xu5647sup1.cif

Structure factors: contains datablock(s) I. DOI: 10.1107/S1600536812046107/xu5647Isup2.hkl

Additional supplementary materials:  crystallographic information; 3D view; checkCIF report

## Figures and Tables

**Table 1:** Hydrogen-bond geometry (Å, °)

*D*—H⋯*A*	*D*—H	H⋯*A*	*D*⋯*A*	*D*—H⋯*A*
O1—H1⋯O2^i^	0.84	1.90	2.740 (6)	173

Symmetry code: (i) 
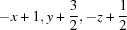
.

**Fig. 1.**
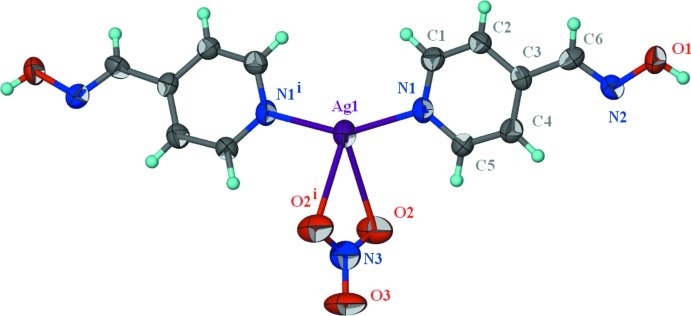
Thermal ellipsoid plot (Barbour, 2001) of Ag(NO3)(C6H6N2O)2 at the 50% probability level; hydrogen atoms are drawn as spheres of arbitrary radius.

**Fig. 2.**
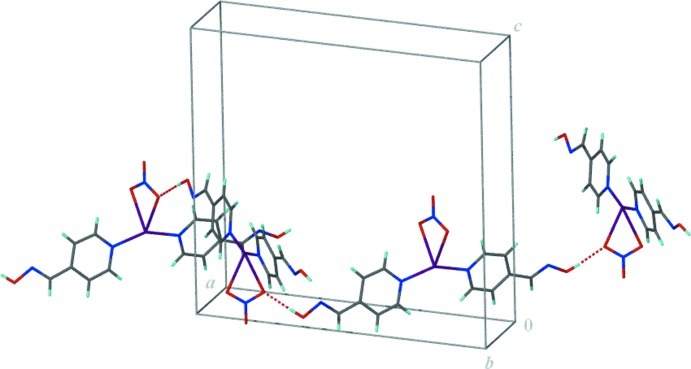
Hydrogen-bonded chain motif.

## supplementary crystallographic information

### Comment

Picolinylaldehyde oxime reacts with silver nitrate to yield a monomeric adduct in which the metal atom is *N*,*N*'-chelated by the ligand. The nitrate ion is also involved in coordination (Abu-Youssef *et al.*, 2010). The corresponding reaction with isonicotinylaldehyde in place of picolinylaldehyde yields a bis adduct (Scheme I). The nitrate-chelated Ag^I^ atom in mononuclear Ag(NO_3_)(C_6_H_6_N_2_O)_2_ is coordinated to the hydroxylamine through its aromatic N atom, and it exists in an approximate tetrahedral geometry (Fig. 1). The hydroxyl OH group forms a hydrogen bond with a nitrate O atom to generate a chain running along the longest axis of the orthorhombic unit cell (Table 1).

### Experimental

Isonicotinaldehyde oxime was synthesized from the reaction of isonicotinaldehyde and hydroxylamine. Silver nitrate (1 mmol) dissolved in water (5 ml) was added to picolinaldehyde oxime (1 mmol) dissolved in ethanol (5 ml). The solution was filtered and set aside, away from light, for the growth of colorless crystals.

### Refinement

Carbon- and oxygen-bound H-atoms were placed in calculated positions (C–H 0.93 Å, O–H 0.84 Å) and were included in the refinement in the riding model approximation, with *U*(H) set to 1.2–1.5*U*(C,*O*).

### Crystal data

**Table:**

[Ag(NO_3_)(C_6_H_6_N_2_O)_2_]	*F*(000) = 824
*M**_r_* = 414.14	*D*_x_ = 1.838 Mg m^−^^3^
Orthorhombic, *P**c**c**n*	Mo *K*α radiation, λ = 0.71073 Å
Hall symbol: -P 2ab 2ac	Cell parameters from 4605 reflections
*a* = 18.027 (3) Å	θ = 3.2–27.5°
*b* = 4.6907 (6) Å	µ = 1.38 mm^−^^1^
*c* = 17.7020 (19) Å	*T* = 293 K
*V* = 1496.9 (3) Å^3^	Prism, colorless
*Z* = 4	0.20 × 0.12 × 0.12 mm

### Data collection

**Table:**

Rigaku R-AXIS RAPID IP diffractometer	1705 independent reflections
Radiation source: fine-focus sealed tube	1038 reflections with *I* > 2σ(*I*)
Graphite monochromator	*R*_int_ = 0.082
ω scan	θ_max_ = 27.5°, θ_min_ = 3.2°
Absorption correction: multi-scan (*ABSCOR*; Higashi, 1995)	*h* = −23→23
*T*_min_ = 0.770, *T*_max_ = 0.852	*k* = −5→6
13223 measured reflections	*l* = −21→22

### Refinement

**Table:**

Refinement on *F*^2^	Secondary atom site location: difference Fourier map
Least-squares matrix: full	Hydrogen site location: inferred from neighbouring sites
*R*[*F*^2^ > 2σ(*F*^2^)] = 0.050	H-atom parameters constrained
*wR*(*F*^2^) = 0.162	*w* = 1/[σ^2^(*F*_o_^2^) + (0.073*P*)^2^ + 1.2436*P*] where *P* = (*F*_o_^2^ + 2*F*_c_^2^)/3
*S* = 1.13	(Δ/σ)_max_ = 0.001
1705 reflections	Δρ_max_ = 0.92 e Å^−^^3^
108 parameters	Δρ_min_ = −0.67 e Å^−^^3^
0 restraints	Extinction correction: *SHELXL97* (Sheldrick, 2008), Fc^*^=kFc[1+0.001xFc^2^λ^3^/sin(2θ)]^-1/4^
Primary atom site location: structure-invariant direct methods	Extinction coefficient: 0.0096 (16)

### Fractional atomic coordinates and isotropic or equivalent isotropic displacement parameters (Å^2^)

**Table:**

	*x*	*y*	*z*	*U*_iso_*/*U*_eq_	
Ag1	0.2500	0.2500	0.18639 (3)	0.0532 (4)	
O1	0.5757 (2)	1.5200 (8)	0.0886 (2)	0.0584 (11)	
H1	0.6143	1.5575	0.1132	0.088*	
O2	0.3058 (3)	0.1732 (13)	0.3234 (2)	0.0789 (15)	
O3	0.2500	0.2500	0.4306 (3)	0.093 (3)	
N1	0.3325 (2)	0.5683 (9)	0.1548 (2)	0.0454 (11)	
N2	0.5350 (3)	1.3109 (9)	0.1264 (2)	0.0487 (11)	
N3	0.2500	0.2500	0.3606 (4)	0.067 (2)	
C1	0.3371 (3)	0.6602 (14)	0.0824 (3)	0.0487 (13)	
H1A	0.3067	0.5752	0.0464	0.058*	
C2	0.3841 (3)	0.8714 (12)	0.0598 (2)	0.0426 (12)	
H2	0.3840	0.9318	0.0098	0.051*	
C3	0.4321 (2)	0.9961 (10)	0.1113 (3)	0.0397 (11)	
C4	0.4286 (3)	0.9016 (12)	0.1858 (3)	0.0466 (13)	
H4	0.4601	0.9782	0.2222	0.056*	
C5	0.3783 (3)	0.6940 (12)	0.2050 (3)	0.0454 (13)	
H5	0.3756	0.6371	0.2552	0.055*	
C6	0.4830 (3)	1.2177 (11)	0.0848 (3)	0.0459 (13)	
H6	0.4771	1.2919	0.0365	0.055*	

### Atomic displacement parameters (Å^2^)

**Table:**

	*U*^11^	*U*^22^	*U*^33^	*U*^12^	*U*^13^	*U*^23^
Ag1	0.0461 (5)	0.0541 (5)	0.0594 (5)	−0.0093 (3)	0.000	0.000
O1	0.051 (2)	0.064 (3)	0.060 (2)	−0.022 (2)	−0.0052 (18)	0.007 (2)
O2	0.054 (3)	0.124 (4)	0.059 (3)	0.037 (3)	0.006 (2)	0.002 (2)
O3	0.084 (6)	0.155 (10)	0.040 (3)	0.050 (5)	0.000	0.000
N1	0.038 (2)	0.047 (3)	0.051 (2)	−0.007 (2)	0.0030 (19)	−0.003 (2)
N2	0.049 (3)	0.050 (3)	0.046 (2)	−0.009 (2)	0.003 (2)	0.0004 (19)
N3	0.057 (5)	0.084 (6)	0.061 (5)	0.020 (4)	0.000	0.000
C1	0.040 (3)	0.061 (3)	0.045 (3)	0.002 (3)	−0.002 (2)	−0.005 (3)
C2	0.039 (3)	0.050 (3)	0.039 (3)	0.000 (3)	0.000 (2)	0.003 (2)
C3	0.034 (2)	0.040 (3)	0.045 (3)	0.002 (2)	0.003 (2)	0.001 (2)
C4	0.047 (3)	0.045 (3)	0.047 (3)	−0.004 (3)	−0.004 (2)	0.000 (2)
C5	0.043 (3)	0.053 (3)	0.041 (3)	−0.009 (2)	−0.001 (2)	−0.002 (2)
C6	0.046 (3)	0.051 (3)	0.041 (2)	0.002 (2)	−0.005 (2)	0.000 (2)

### Geometric parameters (Å, º)

**Table:**

Ag1—N1^i^	2.180 (4)	N3—O2^i^	1.256 (6)
Ag1—N1	2.180 (4)	C1—C2	1.364 (9)
Ag1—O2	2.651 (4)	C1—H1A	0.9300
Ag1—O2^i^	2.651 (4)	C2—C3	1.386 (7)
O1—N2	1.396 (5)	C2—H2	0.9300
O1—H1	0.8400	C3—C4	1.393 (6)
O2—N3	1.256 (6)	C3—C6	1.463 (7)
O3—N3	1.239 (9)	C4—C5	1.374 (8)
N1—C5	1.349 (6)	C4—H4	0.9300
N1—C1	1.354 (6)	C5—H5	0.9300
N2—C6	1.270 (7)	C6—H6	0.9300
			
N1^i^—Ag1—N1	150.3 (2)	C2—C1—H1A	118.4
N1^i^—Ag1—O2	113.62 (18)	C1—C2—C3	120.1 (4)
N1—Ag1—O2	93.94 (18)	C1—C2—H2	119.9
N1^i^—Ag1—O2^i^	93.94 (18)	C3—C2—H2	119.9
N1—Ag1—O2^i^	113.62 (18)	C2—C3—C4	117.4 (4)
O2—Ag1—O2^i^	47.57 (19)	C2—C3—C6	118.7 (4)
N2—O1—H1	109.5	C4—C3—C6	123.9 (4)
N3—O2—Ag1	97.9 (4)	C5—C4—C3	119.3 (5)
C5—N1—C1	116.5 (5)	C5—C4—H4	120.3
C5—N1—Ag1	123.2 (3)	C3—C4—H4	120.3
C1—N1—Ag1	120.2 (3)	N1—C5—C4	123.5 (5)
C6—N2—O1	110.6 (4)	N1—C5—H5	118.3
O3—N3—O2^i^	121.6 (4)	C4—C5—H5	118.3
O3—N3—O2	121.6 (4)	N2—C6—C3	121.4 (5)
O2^i^—N3—O2	116.7 (7)	N2—C6—H6	119.3
N1—C1—C2	123.2 (5)	C3—C6—H6	119.3
N1—C1—H1A	118.4		
			
N1^i^—Ag1—O2—N3	−72.7 (4)	Ag1—N1—C1—C2	175.7 (4)
N1—Ag1—O2—N3	118.8 (3)	N1—C1—C2—C3	2.3 (9)
O2^i^—Ag1—O2—N3	0.000 (1)	C1—C2—C3—C4	−1.5 (8)
N1^i^—Ag1—N1—C5	−171.4 (4)	C1—C2—C3—C6	178.7 (5)
O2—Ag1—N1—C5	−12.8 (4)	C2—C3—C4—C5	−0.5 (8)
O2^i^—Ag1—N1—C5	32.1 (5)	C6—C3—C4—C5	179.4 (5)
N1^i^—Ag1—N1—C1	12.1 (4)	C1—N1—C5—C4	−1.1 (8)
O2—Ag1—N1—C1	170.6 (4)	Ag1—N1—C5—C4	−177.7 (4)
O2^i^—Ag1—N1—C1	−144.4 (4)	C3—C4—C5—N1	1.8 (9)
Ag1—O2—N3—O3	180.0	O1—N2—C6—C3	179.9 (4)
Ag1—O2—N3—O2^i^	0.000 (1)	C2—C3—C6—N2	−170.0 (5)
C5—N1—C1—C2	−1.0 (8)	C4—C3—C6—N2	10.2 (8)

Symmetry code: (i) −*x*+1/2, −*y*+1/2, *z*.

### Hydrogen-bond geometry (Å, º)

**Table:**

*D*—H···*A*	*D*—H	H···*A*	*D*···*A*	*D*—H···*A*
O1—H1···O2^ii^	0.84	1.90	2.740 (6)	173

Symmetry code: (ii) −*x*+1, *y*+3/2, −*z*+1/2.
